# Infection of a French Population of *Aedes albopictus* and of *Aedes aegypti* (Paea Strain) with Zika Virus Reveals Low Transmission Rates to These Vectors’ Saliva

**DOI:** 10.3390/ijms18112384

**Published:** 2017-11-10

**Authors:** Faustine Ryckebusch, Michèle Berthet, Dorothée Missé, Valérie Choumet

**Affiliations:** 1 Environment and Infectious Risks Unit, Institut Pasteur, 75015 Paris, France; faustine.ryckebusch@gmail.com (F.R.); mberthet@pasteur.fr (M.B.); 2 MIVEGEC, Institut de Recherche pour le Développement, CNRS, Univ. Montpellier, 34394 Montpellier, France; dorothee.misse@ird.fr

**Keywords:** Zika virus, *Aedes albopictus*, *Aedes aegypti*, dissemination, transmission, vector competence

## Abstract

Disease caused by the Zika virus (ZIKV) is a public health emergency of international concern. Recent epidemics have emerged in different regions of the world and attest to the ability of the virus to spread wherever its vector, *Aedes* species mosquitoes, can be found. We have compared the transmission of ZIKV by *Ae. aegypti* (PAEA strain originating from Tahiti) and by a French population of *Ae. albopictus* to better assess their competence and the potential risk of the emergence of ZIKV in Europe. We assessed the transmission of ZIKV by *Ae. albopictus* in temperatures similar to those in Southern France during the summer. Our study shows that the extrinsic incubation period of *Ae. aegypti* for transmission was shorter than that of *Ae. albopictus*. Both vectors were able to transmit ZIKV from 10 to 14 days post-infection. *Ae. aegypti*, however, had a longer transmission period than the French population of *Ae. albopictus*. Although the salivary glands of both vectors are highly infected, transmission rates of ZIKV to saliva remain relatively low. These observations may suggest that the risk of emergence of ZIKV in Europe could be low.

## 1. Introduction

Zika virus (ZIKV) is an arthropod-borne virus (arbovirus) in the genus *Flavivirus*, family *Flaviviridae.* Since the 1950s, ZIKV has been reported to have circulated sporadically in Africa and South-East Asia. In 2007, ZIKV was isolated for the first time in the Pacific, on the Micronesian island of Yap [1]. From October 2013 to April 2014, French Polynesia experienced a large Zika outbreak [2,3]. Prior to the French Polynesian outbreak, ZIKV infection was described as a mild febrile illness. It has spread rapidly since 2015 as indigenous cases of the virus have been confirmed in South and Central America, as well as in the Caribbean islands [2,4]. The existence of potential neurological complications and autoimmune diseases has been revealed in recent epidemics. An increase of Guillain-Barré Syndrome cases was observed in French Polynesia in 2013 with a frequency of 2.4 in 10,000 people infected with ZIKV [5]. It is estimated that 40 times more cases of microcephaly have been described in Brazil [6,7,8,9,10,11]. Recent epidemics have emerged in different regions of the world and attest to the ability of the virus to spread wherever vector *Aedes* is located. To date, ZIKV has been isolated from 17 different *Aedes* mosquito species as well as *Culex perfuscus, Mansonia uniformis, Anopheles coustani*, and *Anopheles gambiae* mosquitoes [12,13,14]. In Europe, *Ae. aegypti* is present on the Portuguese island of Madeira [15], as well as in Russia and Georgia [16], and *Ae. albopictus* presents a rapid expanding range in many European countries [17].

We focused our studies on *Aedes aegypti* and *Aedes albopictus* because laboratory studies have shown that these mosquitoes can transmit ZIKV [18,19]. *Ae. aegypti* mosquitoes appear to be the main vectors of ZIKV in Asia [20]. ZIKV has been isolated from this vector in Peninsular Malaysia [21] and Brazil [22]. It is suspected to be the main vector in French Polynesia along with *Aedes polynesiensis* [5]. Recent studies have indicated a relatively low vector competence of American *Ae. aegypti* and *Ae. albopictus* populations [23], as well as that of a French *Ae. albopictus* population [24]. Studies based on the abundance of *Ae. albopictus* in Northern Italy have estimated the R0 of ZIKV to be below the epidemic threshold [25]. We were interested in better analyzing the competence of these two mosquito species. Our approach consisted in comparing the infection of *Ae. aegypti* (PAEA strain originating from Tahiti) and a French population of *Ae. albopictus* (collected in Nice, south-east of France) by ZIKV at the level of the tissue barriers of infection and transmission of arboviruses associated with the midgut and the salivary glands. Contrary to previous studies, we analyzed the infection of the temperate population of *Ae. albopictus* by ZIKV in temperatures similar to those in Southern France during the peak of vector activity.

## 2. Results

### 2.1. Rate of Infection of Ae. aegypti and Ae. albopictus Midguts and Salivary Glands

We used three different techniques to measure and confirm the rates of infection of the midguts (MG) and salivary glands (SG) of *Ae. aegypti* and *Ae. albopictus*. Real time quantitative PCR (RT-qPCR) assessed the replication of the virus in the organs by measuring the number of viral ribonucleic acid (RNA) copies. Titers measured by focus-forming assays (ffa) demonstrated the infectivity of the particles. Western blotting showed the presence of viral proteins. 

**Figure 1:**
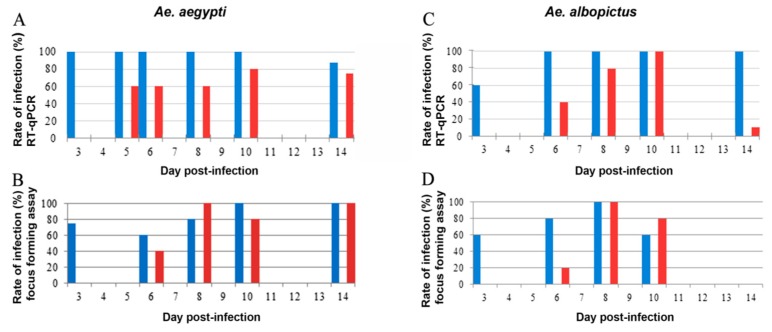
Rate of infection detected by RT-qPCR and immunochemical plaque assay in midguts (MG) and salivary glands (SG) of *Ae. aegypti* and *Ae. albopictus.* MG (blue bars) and SG (red bars) were analyzed by RT-qPCR (**A**,**C**) and titration (**B**,**D**) from *Ae. aegypti* (**A**,**B**) and *Ae. albopictus* (**C**,**D**). *Ae. aegypti* MG and SG were sampled on days 3, 5, 6, 8, 10, and 14, whereas *Ae. albopictus* MG and SG were sampled on days 3, 6, 8, 10, and 14. The graphs present the results of one experiment for which 5 mosquitoes were tested for each time point. It was repeated two times.

**Figure 2:**
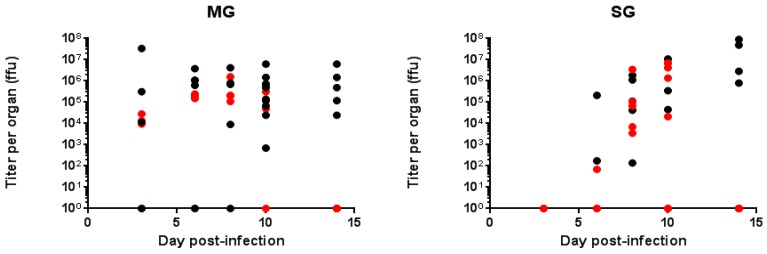
Titration of the Zika virus (ZIKV) in mosquito organs (MG and SG) at different days post-infection. *Ae. aegypti* (black circles) and *Ae. albopictus* (red circles) MG and SG were dissected at various dpi. ffu: focus forming unit.

The results of RT-qPCR (Figure 1A) are in agreement with the titration assays (Figure 1B), indicating that the MG of between 70% and 100% of *Ae. aegypti* were infected within three days post-infection (dpi). We did not find any significant difference in the MG titers between 3 and 14 dpi (Figure 2). Western blots performed at 14 dpi showed the presence of the envelope protein in 90% of the MG samples (data not shown) in agreement with RT-qPCR and titration assays. For *Ae. albopictus*, results obtained by titration and RT-qPCR were consistent and showed that 60% of the MG were infected by ZIKV at 3 dpi (Figure 1C,D). This rate increased to 100% by 6 (RT-qPCR) and 8 dpi (titration), which is similar to *Ae. aegypti*. We did not find any significant difference in *Ae. albopictus* MG titers between 3 and 10 dpi (Figure 2). Contrary to *Ae. aegypti*, we no longer detected ZIKV by titration by 14 dpi in *Ae. albopictus* midguts (Figure 2).

**Figure 3:**
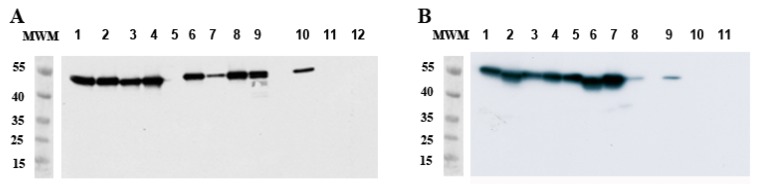
Detection of ZIKV E protein by Western blot in *Ae. aegypti* and *Ae. albopictus* SG at 14 dpi. (**A**) Lines 1 to 9: *Ae. aegypti* SG infected by ZIKV; (**B**) Lines 1 to 8 *Ae. albopictus* SG infected by ZIKV. Controls are shown in each figure and consist of ZIKV-infected Vero cell lysate (positive control, lines 10A and 9B), L15 medium (lines 11A and 10B), and non-infected MG (lines 12A and 11B). MWM: molecular weight markers are indicated in kDa.

RT-qPCR revealed that up to 60% of *Ae. aegypti* SG were infected as early as 5 dpi, showing that the virus quickly spreads to the SG. ZIKV titers in SG were already high at 6 dpi (mean: 2.10 × 10^5^ ffu/organ; SD: 1.05 × 10^5^) and increased at 10 (mean: 4.56 × 10^6^ ffu/organ; SD: 4.57 × 10^6^) and 14 (mean: 5.11 × 10^7^ ffu/organ; SD: 4.51 × 10^7^) dpi. Western blots performed at 14 dpi confirmed data from RT-qPCR and titration, showing the presence of the envelope protein in 88% of the SG samples (Figure 3A). 

Twenty (titration) to forty per cent (RT-qPCR) of *Ae. albopictus* SG were infected at 6 dpi, increasing up to 100% between 8 (titration) and 10 dpi (RT-qPCR). ZIKV titer observed at 6 dpi was low (mean: 7.00 × 10^1^ ffu/organ) and increased at 8 dpi (mean: 7.36 × 10^5^ ffu/organ; SD: 6.88 × 10^5^) to remain stable at 10 dpi (mean: 3.15 × 10^6^ ffu/organ; SD: 2.52 × 10^5^) (Figure 2).

From 8 to 10 dpi, 80 to 100% of SG from both species were found infected (Figure 1).

We did not detect any infectious viral particles in *Ae. albopictus* SG by 14 dpi (Figure 2). Interestingly, we still detected envelope protein (E protein) in 100% of SG at 14 dpi (Figure 3B). Titers measured at 6 dpi in SG differed between the two mosquito species (*p* = 0.001), whereas similar titers were observed at 8 and 10 dpi.

### 2.2. Kinetics of ZIKV RNA Levels in MG and SG of Aedes Mosquitoes

We chose to follow the kinetics of ZIKV infection in MG and SG of *Ae. aegypti* and *Ae. albopictus* by measuring the viral load in MG and SG over time by RT-qPCR assay, a more sensitive technique than titration.

**Figure 4:**
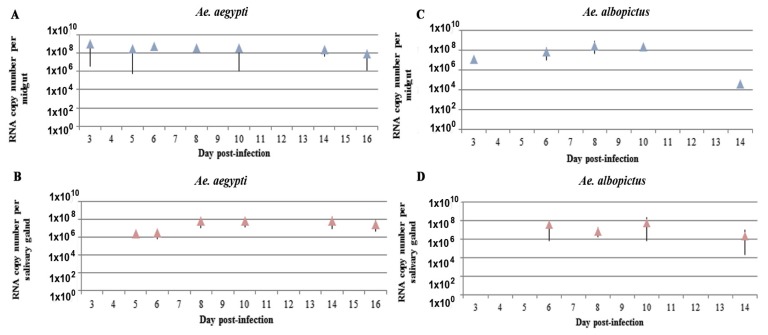
Kinetics of ZIKV infection of MG and SG of *Ae. aegypti* and *Ae. albopictus* measured by RT-qPCR. MG (**A**,**C**) and SG (**B**,**D**) infected by ZIKV in *Ae. aegypti* (**A**,**B**) and *Ae. albopictus* (**C**,**D**). Mean (triangles) and standard deviation (vertical bar) are presented for each organ in both species. Five to ten mosquitoes were sampled for each time.

MG cells of *Ae. aegypti* females were infected by 3 dpi and the viral RNA copy number per organ had already plateaued and remained high out to at least 14 dpi, ranging from 2.3 × 10^8^ to 4.0 × 10^8^ RNA copies (Figure 4A). We did not find any significant difference in the RNA levels between days 3 and 16. The average viral load of ZIKV in the MG of *Ae. albopictus* at 3 dpi was approximately 1.2 × 10^7^ RNA copies. This viral load reached approximately 7.4 × 10^7^ copies of viral RNA by 10 dpi. Again, we did not find any significant difference in the RNA levels between 3 and 10 dpi. ZIKV RNA level decreased significantly to an average of 3.9 × 10^4^ copies of viral RNA by 14 dpi (*p* = 0.002) (Figure 4C). 

Viral replication in *Ae. aegypti* SG started at 5 dpi. Then, from 5 to 16 dpi, we did not observe any significant difference in the RNA levels (Figure 4B). In *Ae. albopictus*, SG RNA levels reached a plateau by 6 dpi with an average of 3.7 × 10^7^ copies of viral RNA detected (Figure 4D). The amount of virus remained stable to 10 dpi and then decreased significantly, falling to 2.1 × 10^6^ copies of viral RNA by 14 dpi (*p* = 0.008).

The level of viral RNA of the MG of these two species differed: at 6 dpi, the MG of *Ae. aegypti* contained higher amounts than that of *Ae. albopictus* (*p* = 0.008) (Figure 4A,C). The SG of *Ae. aegypti* were also more highly infected at 8 dpi than those of *Ae. albopictus* (*p* = 0.038) (Figure 4B,D).

We detected significantly greater infection of the MG (*p* = 0.0045) and SG (*p* = 0.004) of *Ae. aegypti* than *Ae. albopictus* at 14 dpi, characterized by a lower amount of viral RNA in these two organs in *Ae. albopictus*. A comparison of the titration and RT-qPCR assays showed that at 14 dpi, we still detected ZIKV viral RNA in the SG of *Ae. albopictus* (Figure 4D), whereas no infectious particles were detected (Figure 1D). Apart from these differences, the results obtained by RT-qPCR in the MG and SG of *Ae. aegypti* and *Ae. albopictus* (Figure 4) were in agreement with those of the titration assay (Figure 1B,D). 

**Figure 5:**
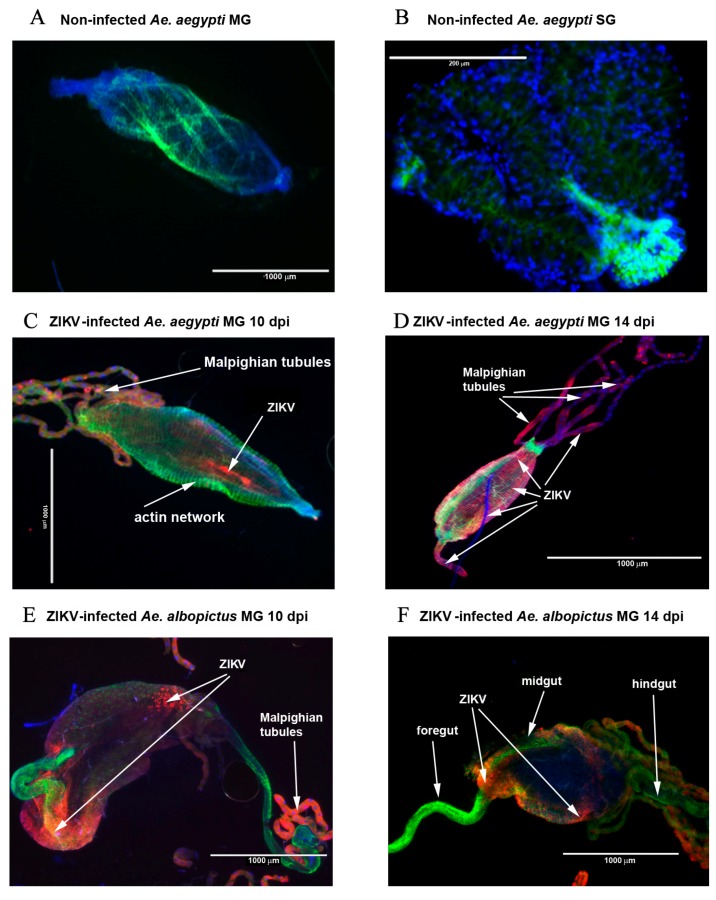

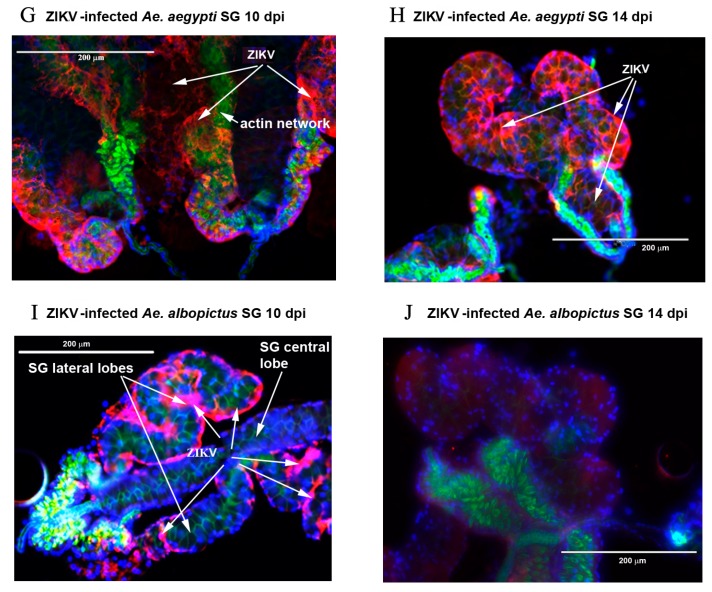
Dynamics of ZIKV infection in MG and SG of *Ae. aegypti* and *Ae. albopictus* at 10 and 14 dpi by microscopy. Nuclei are labeled in blue, E protein of ZIKV in red, and the actin network in green. (**A**) non-infected MG of *Ae. aegypti* at 14 dpi; (**B**) Non-infected SG of *Ae. aegypti* at 14 dpi; (**C**,**D**) *Ae. aegypti* MG infected by ZIKV at 10 and 14 dpi, respectively; (**E**,**F**) *Ae. albopictus* MG infected by ZIKV at 10 and 14 dpi, respectively; (**G**,**H**) *Ae. aegypti* SG infected by ZIKV at 10 and 14 dpi, respectively; (**I**,**J**) *Ae. albopictus* SG infected by ZIKV at 10 and 14 dpi, respectively.

We also visualized ZIKV envelope protein (E protein) in both organs by immunofluorescence at 10 and 14 dpi. Figure 5 shows the distribution of ZIKV antigens in the SG and MG of *Ae. aegypti* and *Ae. albopictus* at 10 and 14 dpi. We detected viral antigens in the MG in highly localized foci at 10 dpi (Figure 5C,E), with an axial localization for *Ae. aegypti* (Figure 5C) and at the posterior and anterior parts of MG for *Ae. albopictus* (Figure 5E). Viral antigens then became evenly distributed over the entire epithelium by 14 dpi, especially for *Ae. aegypti* (Figure 5D). The foregut and hindgut did not appear to be infected in *Ae. albopictus* (Figure 5E,F), while we can see viral antigens in the foregut of *Ae. aegypti* at 14 dpi (Figure 5D). The SG were mostly infected at 10 and 14 dpi in the proximal and distal region of the lateral and central lobes in *Ae. aegypti* mosquitoes (Figure 5G,H). Lateral SG lobes were found infected in *Ae. albopictus* mosquitoes at 10 dpi (Figure 5I), whereas only a faint labeling was observed at 14 dpi (Figure 5J). The Malpighian tubules were also infected with ZIKV both in *Ae. albopictus* and *Ae. aegypti* (Figure 5D,E). 

### 2.3. Transmission Rate of ZIKV in Aedes Saliva

We then determined whether infected mosquitoes were able to transmit ZIKV. For this purpose, mosquitoes were anesthetized, legs were removed, and their saliva was collected in a small amount of medium. We then analyzed the saliva of the mosquitoes by dot-blot to determine both the presence of saliva (using rabbit anti-saliva antibodies) and ZIKV (using anti-E 4G2 monoclonal antibodies). All mosquitoes do not salivate under these conditions, potentially leading to an underestimation of the number of ZIKV-positive saliva samples in other reports. We then quantified ZIKV in saliva-positive samples by RT-qPCR. As already discussed, we also quantified ZIKV in mosquitoes after saliva collection to investigate their infection status.

We detected ZIKV by 8 dpi in the saliva of *Ae. aegypti*, with 11% of the saliva samples found to be infected. The proportion of infected saliva samples increased three fold by two days later and then decreased from 14 to 17 dpi (Table 1). The RNA copy number increased from 8 dpi to reach a maximum at 10 dpi and remained stable until 17 dpi (Table 1). 

We detected ZIKV-positive saliva for *Ae. albopictus* at 10 dpi with a rate of infection of 28% (Table 1). The proportion of infected saliva remained stable until 14 dpi. The viral RNA copy number did not differ significantly between 10 and 14 dpi. The viral load of *Ae. aegypti* saliva at 10 and 14 dpi was significantly higher than that of *Ae. albopictus* saliva (*p* = 0.007).

We determined whether the viral load of the whole mosquito influenced the proportion of ZIKV-positive saliva at 10 and 14 dpi. We quantified ZIKV in all mosquitoes used for salivation by RT-qPCR (Table 2). We found no difference between viral RNA levels of saliva-positive and saliva-negative total mosquitoes for *Ae. aegypti*, whereas *Ae. albopictus* mosquitoes with higher viral loads had a higher proportion of ZIKV-positive saliva at 14 dpi (*p* = 0.03).

### 2.4. Analysis of Viral Particles in Both Mosquito Species

Our results showed that we were still able to measure viral RNA in *Ae. albopictus* SG at 14 dpi, while no infectious viral particles could be detected. We were therefore interested in analyzing the viral copy/ffu ratio of ZIKV in cell culture and in the MG and SG of *Ae. aegypti* and *Ae. albopictus*. We also determined whether viral protein degradation could be observed in the infected MG and SG extracts. 

#### 2.4.1. Quantification of Defective ZIKV Particles in SG and MG of *Ae. aegypti* and *Ae. albopictus* at Different Time Points

We measured the proportion of defective particles in MG and SG of the two mosquito species at different times post infection, based on the number of defective viral particles from the viral stock produced in C6/36 cells. We calculated the ratio of the number of viral RNA copies/ml found by RT-qPCR and the number of focus forming units, resulting in a value of 39 RNA copies per infectious particle for the virus in culture.

Both species had a higher number of defective particles in the MG than SG (Table 3). This was particularly true for *Ae. albopictus,* for which we found an average of 10 to 16 times more defective particles in the MG than after amplification in C6/36 cells. The number of defective particles did not differ greatly depending on the number of dpi in *Ae. aegypti* MG, while in *Ae. albopictus* this number was more variable according to the number of dpi (Table 3).

#### 2.4.2. Degradation of Viral Proteins

**Figure 6:**
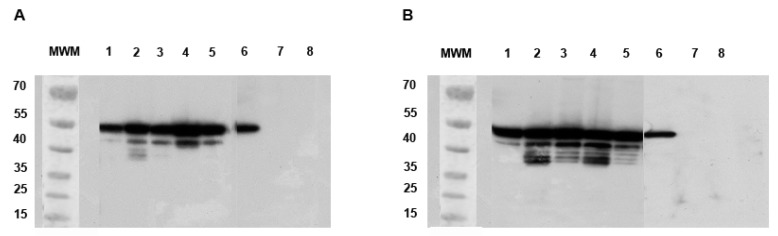
Western blots comparing the degradation of ZIKV E protein in *Ae. aegypti* and *Ae. albopictus* at 10 dpi. (**A**) Lines 1 to 5: *Ae. aegypti* MG infected by ZIKV; (**B**) Lines 1 to 5: *Ae. albopictus* MG infected by ZIKV. Controls are shown in each figure and consist of ZIKV-infected Vero cell lysate (positive control, lines 6A and B), L15 medium (lines 7A and B), and non-infected MG (lines 8A and B). MWM: molecular weight markers are indicated in kDa.

We analyzed MG by western blotting at 10 dpi. We found a major band at 53 kDa, corresponding to the normal size of the ZIKV envelope protein (Figure 6) [26]. A degraded form of approximately 45 kDa was also prevalent in the MG of *Ae. aegypti* (Figure 6A), whereas many bands of the envelope protein between 35 and 45 kDa were found in the MG of *Ae. albopictus* (Figure 6B). These bands were not observed in non-infected MG (Figure 6A,B, lines 8). The profiles observed in SG dissected at 10 dpi are similar to those observed at 14 dpi (see Figure 3).

## 3. Discussion

Since its first isolation in the Zika forest in 1947, ZIKV has caused a self-limiting febrile illness from Africa to South-East Asia for decades [20,27,28,29]. It has recently spread across the Pacific where it has caused two major epidemics, one in Yap Island in 2007 and the other in French Polynesia in 2013–2014 [1,30]. In 2015, this virus caused infections for the first time in South America [31]. Although perinatal and sexual routes of infection have been identified for ZIKV [32,33], this arbovirus is primarily transmitted by mosquito vectors. *Ae. aegypti* and *Ae. albopictus* are vectors of globally important arboviruses, such as dengue and chikungunya viruses [34]. In Africa, ZIKV has been isolated from *Ae. aegypti* in Ivory Coast [35] and a high prevalence of antibodies against this virus was found in Nigeria, suggesting that the mosquito *Ae. aegypti* plays a role in the urban transmission of this virus. *Ae. albopictus* mosquitoes may have been the drivers of the epidemic in Gabon in 2007, as shown by virus isolation [36]. The range of *Ae. albopictus* extends to all continents, including Europe, where it is now installed in many countries [37]. This species has been demonstrated to transmit ZIKV both in the laboratory [23,24,38,39] and in the wild [36].

Our study shows that the kinetics of infection as well as rates of infection are similar between the two vectors until 10 dpi. However, a major difference in the kinetics of infection was observed at 14 dpi. We show a persistence of infectious viral particles in the MG and SG of *Ae. aegypti* that we did not find in the French population of *Ae. albopictus*. We also observed a significantly higher degradation of ZIKV E protein in the MG of *Ae. albopictus* than in those of *Ae. aegypti* at 10 dpi. This may explain why we no longer detected infectious viral particles at 14 dpi in *Ae albopictus*. Immunofluorescence showed localization of viral antigens in MG and SG of *Ae aegypti.* It was localized in foci in the MG at early time points in both mosquito species. Both lateral and central lobes of *Ae. aegypti* SG were infected at 10 dpi, whereas viral antigens were only detected in lateral lobes of the SG of *Ae. albopictus*. The Malpighian tubules were infected in both species, as previously reported by Salazar et al. [40] in case of dengue serotype 2 infection. Moreover, the level of ZIKV RNA was significantly higher in *Ae. aegypti* than in *Ae. albopictus,* both in the MG at 6 dpi and in SG at 8 dpi. 

Both vectors were able to transmit ZIKV from 10 to 14 dpi. Our study shows also that ZIKV has a shorter extrinsic incubation period in *Ae. aegypti* (8 days) than in the French population of *Ae. albopictus* (10 days). Thus, *Ae. albopictus* would have less time and fewer opportunities than *Ae. aegypti* to transmit the virus during a blood meal. The infection rates of saliva were similar at 10 and 14 dpi for the two vectors and the mean viral concentration did not vary between these two time points. However, the viral concentration of ZIKV was lower in the saliva of *Ae. albopictus* than that in *Ae. aegypti*. We also observed that only the most infected *Ae. albopictus* mosquitoes seemed to have virus in their saliva at 14 dpi.

Geographic variations in the vector competence of mosquito species for various viruses have been documented, especially for DENV in *Ae. aegypti* [41] and *Ae. albopictus* [42]. Our results obtained with the French population of *Ae. albopictus* are markedly different from those of a study of vector competence of *Ae. albopictus* from Singapore to an African genotype of ZIKV [43]. In the latter, all mosquitoes exhibited infected saliva at 10 dpi. The same study included *Ae. aegypti* from Singapore, and our results are similar to theirs. Studies have been recently published using the Asian genotype of ZIKV served to infect *Ae. aegypti* and *Ae. albopictus* from the Caribbean or Europe [23,24]. These studies revealed similar rates of infection and dissemination as our study, but a markedly lower transmission rate. This difference may be due to the fact that the presence of mosquito saliva in the medium where mosquitoes have been salivating was not tested before ZIKV detection or to the fact that mosquitoes were maintained under tropical conditions that are not appropriate for temperate countries. Indeed, Vega-Rua et al. [44] showed that temperate mosquitoes behave differently compared to their counterpart from tropical regions.

## 4. Materials and Methods

### 4.1. Viral Strain

Mosquitoes were orally exposed to the PF-25013-18 strain of ZIKV (obtained via V. M. Cao-Lormeau and D. Musso, Institut Louis Malardé, Papeete, French Polynesia). This strain was isolated from a viremic patient in French Polynesia in 2013 [45]. ZIKV was first amplified on Vero E6 cells, and for our experiments it was grown in *A. albopictus* C6/36 mosquito cell line.

### 4.2. Mosquito Population

We used an *Ae. albopictus* population that comes from mosquitoes caught in Nice (South of France) in 2011 and the Paea strain of *Ae. aegypti*, a laboratory colony originated from mosquitoes collected in French Polynesia in 1994 and conserved in the laboratory for 400–450 generations. Adult mosquitoes were maintained at 25 ± 1 °C and 80% relative humidity with a light/dark ratio of 12 h/12 h. The larvae were provided with brewer’s yeast tablets and adults were given continuous access to 10% sucrose solution. 

### 4.3. Oral Infection of Mosquitoes and Dissections

Sucrose was removed 24 h prior to the infectious blood meal. Seven day-old female mosquitoes were then allowed to feed for 15 min through a chicken skin covering glass feeders (*Ae. albopictus*) or through a collagen membrane covering electric feeders (*Ae. aegypti*) maintained at 37 °C. The infectious blood meal was comprised of half-washed rabbit erythrocytes and half-viral suspension (2.5 × 10^7^ ffu/mL in the mix), as well as ATP (as a phagostimulant) at a final concentration of 5 × 10^3^ M. Blood-fed females were selected and transferred into cardboard boxes protected with mosquito nets. After feeding, blood-fed mosquitoes were kept at 27 ± 1 °C for *Ae. aegypti* or with a temperature gradient from 22 °C (night) to 25 °C (day) for *Ae. albopictus*, a 16 h:8 h light:dark cycle, and 80% humidity. Mosquitoes were dissected at various time points after oral exposure. Experiments were reproduced in triplicate with 5–10 mosquitoes collected at each time point for dissection.

### 4.4. Mosquito Salivation

ZIKV infected mosquitoes were chilled, and legs and wings were detached. The proboscis was introduced into a 10 µL low binding pipette tip filled with 5 µL filtered phosphate buffer saline (PBS) or DMEM + Glutamax (Dulbecco) containing 2% fetal bovine serum (FBS). The tip contents were collected 30 min later in a tube. Two microliters were transferred to a tube containing 2 µL radioimmunoprecipitation assay (RIPA) lysis buffer and analyzed by dot-blot to verify the presence of saliva and ZIKV. Four microliters were analyzed by RT-qPCR and 4 µL by plaque assay to determine virus titer. Saliva was collected at three time points: 8, 10, and 14 days after exposure to the infectious blood-meal. Ten to 20 mosquitoes were analyzed for each time point and experiments were reproduced three times.

### 4.5. Focus Forming Assay

ZIKV-containing samples were titrated on African green monkey kidney cells (Vero E6) by the plaque assay method. Ten-fold serial dilutions of each sample were deposited in each well of a 24-well plate for 1 h at 37 °C in a CO_2_ incubator. Then, carboxymethyl cellulose-containing medium was deposited into each well and the cells incubated for three days. The medium was removed and the wells washed with PBS. Cells were fixed with a 4% paraformaldehyde solution for 20 min and then with PBS. After washing, the cells were permeabilized in 0.1% triton X-100 for 5 min at RT. After washing with PBS, the cells were incubated with a 1/1000 dilution of 4G2 primary antibody for 30 min. After washing, a 1/500 dilution of anti-mouse HRP secondary antibody was added to each well. Following incubation, the wells were washed in PBS and the Vector VIP kit (Vector Laboratories, Burlingame, CA, USA) used to visualize Focus Forming Units.

### 4.6. RT-qPCR

Synthetic RNA transcripts for ZIKV were generated to construct a standard curve. The targeted region in the ZIKV nucleotide sequence (*NS5* protein gene) was amplified by PCR and ligated into the pUC57 plasmid. The plasmid was then linearized using ScaI restriction enzyme and purified using the Endofree Plasmid Maxi Kit (Qiagen, Foster City, CA, USA). The linearized DNA template was extracted using phenol/chloroform. RNA transcripts were prepared in vitro using the MEGAscript T7 Kit (Thermofisher Scientific, Villebon-sur-Yvette, France). Residual DNA was eliminated by TURBO DNase contained in the kit. After spectrophotometric quantification and electrophoresis, RNA transcripts were kept at −80 °C. The Power SYBR Green RNA-to-Ct One-Step Kit (Applied Biosystems, Foster City, CA, USA) was used following the manufacturer’s procedure for RT-qPCR. The primers were designed to amplify a 101 bp sequence located between nucleotide 9419 and nucleotide 9318 of the *NS5* segment of ZIKV and were as follows: upper 5′-AAGTACACATACCAAAACAAAGTG-3′ and lower 5′-TCCGCTCCCCCTTTGGTCTTG-3′. Duplicates of mosquito samples were analyzed against a standard curve produced using a specific range of synthetic RNA concentration. Samples were amplified on an Applied Biosystems 7500 instrument using the following PCR program: a first step of reverse transcriptase (30 min at 48 °C); a second step of inactivation of the reverse transcriptase and activation of DNA polymerase for 10 min at 95 °C; then, 40 PCR cycles of 15 s at 95 °C and 1 min at 60 °C were performed, during which fluorescence data was collected; and finally, 20 s at 95 °C with ramping of 19 min 59 s for melting curve calculation. 

### 4.7. Immunofluorescence

After dissection, MG were deposited on slides and fixed in acetone for 15 min. SG were fixed on a slide in 4% paraformaldehyde for 15 min. After drying, they were stored at 4 °C until use. For indirect fluorescent-antibody experiments, the MG and SG were rehydrated in PBS for 15 min. The MG were then incubated for 2 h and SG for 15 min in Triton X100 (0.2%). After washing with PBS, they were incubated for 30 min with PBS + 0.1% Tween 20 + 1% BSA. The slides were then incubated overnight at 4 °C with anti-flavivirus E protein 4G2 diluted 1:1000 in PBS. After washing with PBS (3 times × 5 min/time), they were incubated for 1 h with 1:500 Cy5 goat anti/mouse (Invitrogen, Paris, France) and washed with PBS. We used phalloidin Alexafluor 488 (Invitrogen) to visualize the actin network. After washing, nuclei were stained using Prolong gold antifade containing 4′,6-diamidino-2-phenylindole (DAPI) (Invitrogen). A coverslide was added. All preparations were observed by fluorescence microscopy.

### 4.8. Western Blot

Samples (individual MG and SG) were collected in RIPA buffer added with protease inhibitors. They were run on a 4–12% NuPAGE Bis-Tris gel for 1 h at 150 V per gel. The proteins were then transferred to PVDF membranes (TransBlot Turbo, BioRad, Marne-la-Coquette, France). Membranes were blocked with 5% milk in 1% PBS for 1 h at RT and then incubated with the 4G2 antibody overnight at 4 °C. After washing, they were incubated with anti-mouse HRP antibody for 1 h at RT. The blots were incubated with ECL substrate (Amersham, Piscataway, NJ, USA) for 5 min at RT and then revealed on Kodak film.

### 4.9. Dot-Blot

The saliva from each mosquito collected on 8, 10, and 14 dpi in RIPA was blotted onto PVDV membranes. Positive (*Ae. aegypti* salivary gland extract) and negative controls (Leibowitz L15 medium) were then deposited on the membrane. The membranes were blocked as indicated for western blots and incubated for 1 h at RT with an in-house rabbit anti-*Aedes* saliva or 4G2 monoclonal antibody. After washing, the membranes were incubated with anti-mouse or anti-rabbit HRP-labelled antibodies for 1 h at RT. The blots were then revealed as indicated above.

### 4.10. Statistical Analyses

The Simstat software was used for all analyses that were performed using Kruskal Wallis and Mann–Whitney non-parametric tests.

## 5. Conclusions

In conclusion, our results indicate that *Ae. aegypti* are likely more competent for the transmission of ZIKV than the French *Ae. albopictus* population. These observations may suggest that the risk of emergence of ZIKV in Europe could be low. Interestingly, the transmission rates of this virus to the saliva of both *Ae. aegypti* and *albopictus* were relatively low, whereas the SG were highly infected. This suggests that there may be a transmission barrier between SG and saliva. Other factors, such as the close proximity of the vector to humans, the large number of mosquitoes, or possible vertical transmission [46,47], may explain the current epidemic in South America and enhance the risk of emergence in Europe [39]. 

## Figures and Tables

**Table 1:** Rate of transmission of ZIKV by *Aedes* mosquitoes at different times post-infection.

Day Post-Infection	8	10	14	17
*Ae. aegypti*				
% infection	11	33	16	6.7
RNA copy number mean	7.4 × 10^4^	3.2 × 10^5^	1.1 × 10^5^	1.6 × 10^5^
Standard error	-	2.1 × 10^5^	6.8 × 10^4^	-
Number of saliva tested	9	9	25	15
*Ae. albopictus*				
% infection	0	28.6	25	Not collected
RNA copy number mean		4.5 × 10^4^	5 × 10^4^	
Standard error		2.6 × 10^4^	3 × 10^4^	
Number of saliva tested	9	9	16	

**Table 2:** Viral RNA levels detected in *Ae. aegypti* and *Ae. albopictus* mosquitoes according to the presence/absence of ZIKV in their saliva.

Day Post-Infection	8	8	10	10	14	14
	ZIKV − Saliva	ZIKV + Saliva	ZIKV − Saliva	ZIKV + Saliva	ZIKV − Saliva	ZIKV + Saliva
RNA copy number per total *Ae. aegypti* mosquito						
Mean	3.4 × 10^7^	2.5 × 10^7^	8.0 × 10^7^	5.2 × 10^7^	1.3 × 10^8^	2.5 × 10^8^
SD	7.6 × 10^7^	-	6.1 × 10^7^	4.5 × 10^7^	1.9 × 10^8^	1.5 × 10^8^
*N*	8	1	7	3	2	4
RNA copy number per total *Ae. albopictus* mosquito						
Mean	6.1 × 10^7^	-	8.8 × 10^7^	2.1 × 10^8^	2.1 × 10^8^ *	9.4 × 10^8^ *
SD	7.6 × 10^7^	-	6.1 × 10^7^	2.5 × 10^8^	1.9 × 10^8^	8.32 × 10^8^
*N*	8		4	2	5	6

ZIKV **−** Saliva: mosquito with ZIKV-negative saliva; mosquito with ZIKV + Saliva: ZIKV-positive saliva; * significant difference; *N*: number of sampled mosquitoes.

**Table 3:** Viral copy/ffu ratio in *Ae. aegypti* and *Ae. albopictus* midguts and salivary glands.

Day Post-Infection	*Ae. aegypti*				*Ae. albopictus*			
	Midguts		Salivary Glands		Midguts		Salivary Glands	
	Viral Copy/ffu	*R*	Viral Copy/ffu	*R*	Viral Copy:ffu	*R*	Viral Copy/ffu	*R*
3	1.16 × 10^2^	3	-		4.26 × 10^2^	10.9	-	
6	2.46 × 10^2^	6.3	1.48 × 10^1^	0.4	1.63 × 10^2^	4.2	1.5 × 10^2^	3.8
8	2.31 × 10^2^	5.9	1.07 × 10^2^	2.7	3.98 × 10^2^	10.2	8.80	0.2
10	1.31 × 10^2^	3.3	1.63 × 10^1^	0.4	6.47 × 10^2^	16.6	1.81 × 10^1^	0.5
14	1.49 × 10^2^	3.8	1.67	0.04	-		-	

*R*: The ratios between the number of copies of viral RNA and the number of infectious viral particles in mosquito organs were divided by the ratio calculated for ZIKV produced in C6/36 cells and are presented in each column; -: no infectious viral particles were detected.

## Abbreviations

MG	Midgut
SG	Salivary gland
ffa	Focus forming assay
ffu	Focus forming unit
dpi	Day post-infection
